# The pathway intermediate 2‐keto‐3‐deoxy‐L‐galactonate mediates the induction of genes involved in D‐galacturonic acid utilization in *Aspergillus niger*

**DOI:** 10.1002/1873-3468.12654

**Published:** 2017-05-06

**Authors:** Ebru Alazi, Claire Khosravi, Tim G. Homan, Saskia du Pré, Mark Arentshorst, Marcos Di Falco, Thi T. M. Pham, Mao Peng, Maria Victoria Aguilar‐Pontes, Jaap Visser, Adrian Tsang, Ronald P. de Vries, Arthur F. J. Ram

**Affiliations:** ^1^ Molecular Microbiology and BiotechnologyInstitute of Biology LeidenLeiden UniversityThe Netherlands; ^2^ Fungal PhysiologyWesterdijk Fungal Biodiversity InstituteUtrecht UniversityThe Netherlands; ^3^ Centre for Structural and Functional GenomicsConcordia UniversityMontrealCanada

**Keywords:** d‐galacturonic acid catabolism, gene regulation, pectinase

## Abstract

In *Aspergillus niger*, the enzymes encoded by *gaaA*,* gaaB*, and *gaaC* catabolize d‐galacturonic acid (GA) consecutively into l‐galactonate, 2‐keto‐3‐deoxy‐l‐galactonate, pyruvate, and l‐glyceraldehyde, while GaaD converts l‐glyceraldehyde to glycerol. Deletion of *gaaB* or *gaaC* results in severely impaired growth on GA and accumulation of l‐galactonate and 2‐keto‐3‐deoxy‐l‐galactonate, respectively. Expression levels of GA‐responsive genes are specifically elevated in the *∆gaaC* mutant on GA as compared to the reference strain and other GA catabolic pathway deletion mutants. This indicates that 2‐keto‐3‐deoxy‐l‐galactonate is the inducer of genes required for GA utilization.

## Abbreviations

**AP**, apple pectin

**CM**, complete medium

**GA**, d‐galacturonic acid

**MM**, minimal medium

**NMR**, Nuclear Magnetic Resonance Spectroscopy

**PGA**, polygalacturonic acid

**RG‐I**, rhamnogalacturonan I

**α‐IPM**, α‐isopropylmalate

**Figure 1:**
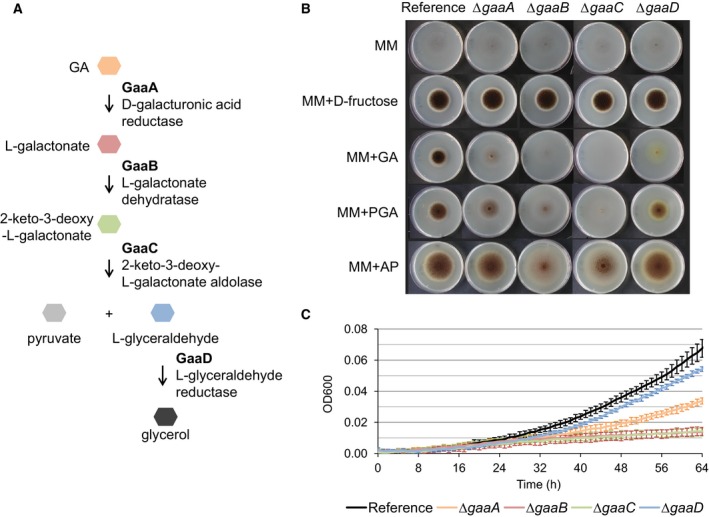
(A) The evolutionarily conserved GA catabolic pathway in filamentous fungi as proposed by Martens‐Uzunova and Schaap 5. GA is converted in pyruvate and glycerol by consecutive action of GaaA, GaaB, GaaC, and GaaD enzymes. Growth profile of the reference strain (MA249.1) and GA catabolic pathway deletion mutants *∆gaaA*,* ∆gaaB*,* ∆gaaC, and ∆gaaD* (B) on solid MM without any carbon source, or with 50 mm monomeric or 1% polymeric carbon sources after 7 days at 30 °C, and (C) in microtiter plate in liquid medium with 50 mm GA at 30 °C. Error bars represent standard deviation of six biological replicates.

Pectins are heterogeneous plant cell wall polysaccharides rich in d‐galacturonic acid (GA). They represent a natural carbon source for many saprotrophic fungi including *Aspergillus niger* 1, 2. The *A. niger* genome contains 58 genes encoding pectin‐degrading enzymes 2, 3. GA, the most abundant uronic acid in pectin, is transported by *A. niger* into the cell *via* the transporter GatA 4 and then catabolized into pyruvate and glycerol by consecutive action of four enzymes: GaaA, d‐galacturonate reductase; GaaB, l‐galactonate dehydratase; GaaC, 2‐keto‐3‐deoxy‐l‐galactonate aldolase; and GaaD, l‐glyceraldehyde reductase 5, 6, 7, 8 (Fig. 1A). This four‐step GA catabolic pathway is evolutionarily conserved in Pezizomycotina fungi 5, and has been studied in detail in *Botrytis cinerea* 9 and *Trichoderma reesei* 10, 11, 12, 13. In *B. cinerea,* the first enzymatic step is catalyzed by two functionally redundant enzymes, BcGar1 and the *A. niger* GaaA ortholog BcGar2 9. In *T. reesei*, GA is converted into l‐galactonate by TrGar1 10. In addition, GaaA and GaaD (LarA) of *A. niger* have been shown to be involved in d‐glucuronate and l‐arabinose catabolism, respectively 14, 15.

Degradation of plant cell wall polysaccharides and subsequent transport and catabolism of released sugars are tightly controlled 16. Genes required for pectin degradation, GA transport, and GA catabolism are subject to carbon catabolite repression *via* CreA 17, 18. They are specifically induced in the presence of GA 5, 17 and are regulated by the GaaR/GaaX activator‐repressor module 19, 20. The conserved Zn(II)2Cys6 transcription factor GaaR is required for growth on GA and for the activation of the GA‐responsive genes in both *B. cinerea* and *A. niger* 19, 21.

The mechanism of activation of transcription factors can be diverse, and possibly requires so‐called inducer molecules. These inducer molecules are often metabolites related to the substrate 22. Only a few examples of activation of a transcription factor *via* an inducer have been elucidated in fungi. Probably the best studied example is the Zn(II)2Cys6 transcription factor Gal4p that regulates galactose utilization in *Saccharomyces cerevisiae*. Gal4p is repressed under noninducing conditions because the transcriptional activation domain of Gal4p is bound to the corepressor Gal80p. In the presence of galactose and ATP (inducing conditions), the sensor protein Gal3p binds to the Gal4p/Gal80p complex leading to dissociation of Gal4p and subsequent Gal4p‐dependent transcription 23, 24, 25, 26, 27. In the regulation of leucine biosynthesis, the Zn(II)2Cys6 transcription factor Leu3p interacts directly with a metabolic intermediate. The middle domain of the Leu3p protein masks the C‐terminal activation domain by an intramolecular interaction in the absence of α‐isopropylmalate (α‐IPM), a metabolic intermediate of the leucine biosynthesis pathway. In the presence of α‐IPM, which accumulates during leucine starvation, this self‐masking is prevented, resulting in active Leu3p and activation of leucine biosynthesis genes 28, 29, 30. The Gal4p and Leu3p transcription factors localize to the nucleus regardless of the presence or absence of inducer molecules 31, 32. On the other hand, the transcriptional activator AmyR, involved in starch degradation in *Aspergillus nidulans* and *Aspergillus oryzae*, is translocated from the cytoplasm to the nucleus only in the presence of its inducer isomaltose 33, 34, 35.

In *A. niger*, GA or a derivative of GA was suggested to act as an inducer required for the activation of GA‐responsive genes 17. In *B. cinerea*, BcGaaR was shown to translocate from the cytoplasm to the nucleus in response to such an inducer 21. Previous studies of *A. niger* and *B. cinerea* mutants disrupted in GA catabolic pathway did not unambiguously identify a specific inducer 6, 7, 8, 9. In this study, we constructed GA catabolic pathway deletion mutants *(∆gaaA*,* ∆gaaB*,* ∆gaaC*, and *∆gaaD*) to gain insight into regulation of GA‐responsive genes in *A. niger*. Comparative analysis of these mutants indicates that 2‐keto‐3‐deoxy‐l‐galactonate acts as the physiological inducer of the GA‐responsive genes.

## Materials and methods

### Strains, media and growth conditions

All strains used in this study are listed in Table S1. MA249.1 was obtained by transformation of N593.20 (*cspA1, pyrG* ^*‐*^ *, kusA::amdS*) 19 with a 3.8‐kb *Xba*I fragment containing the *A. niger pyrG* gene, resulting in the full restoration of the *pyrG* locus.

Media were prepared as described previously 36. Radial growth phenotype analyses were performed with minimal medium (MM) (pH 5.8) containing 1.5% (w/v) agar (Scharlau, Barcelona, Spain) and various carbon sources: 50 mm glucose (VWR International, Amsterdam, the Netherlands), d‐fructose (Sigma‐Aldrich, Zwijndrecht, the Netherlands), GA (Chemodex, St Gallen, Switzerland), l‐rhamnose (Fluka, Zwijndrecht, the Netherlands), l‐arabinose (Sigma‐Aldrich) or glycerol (Glycerol 87%; BioChemica AppliChem, Darmstadt, Germany), or 1% (w/v) polygalacturonic acid (PGA) (Sigma), apple pectin (AP) (Sigma‐Aldrich), or galactan (Acros Organics, Geel, Belgium). Filter sterilized d‐fructose or GA solution was added after autoclaving MM with agar. Other carbon sources were autoclaved together with the medium. The plates were inoculated with 5 μL 0.9% NaCl containing 10^4^ freshly harvested spores and cultivated at 30 °C for 7 days. For microtiter plate growth phenotype analysis, wells in a 96‐well, flat bottom plate (Sarstedt AG & Co., Nümbrecht, Germany) were filled with 180 μL MM (pH 5.8) containing 55 mm GA as the sole carbon source, and 20 μL freshly harvested spores (7.5 × 10^5^ spores·mL^−1^). The plate was incubated with lids in EnSpire Multimode Plate Reader (PerkinElmer, Waltham, MA, USA) at 30 °C. Lid temperature was set to 32 °C to prevent condensation on the lid. Optical density at 600 nm was measured every hour. The average OD from the GA‐containing control wells was subtracted from the OD of the test wells and negative values were corrected as zero.

For gene expression and metabolic analyses, 10^8^ freshly harvested spores were inoculated and grown in 100 mL complete medium (CM) (pH 5.8) with 2% (w/v) d‐fructose for 16 h, and mycelia were harvested by filtration through sterile myracloth. For northern blot and metabolic analyses, pregrown mycelia were washed twice with MM with no carbon source (pH 4.5) and 1.5 g (wet weight) mycelia were transferred and incubated in 50 mL MM (pH 4.5) with 50 mm d‐fructose or 50 mm GA for 2 h. For metabolic analysis, 1.5 g (wet weight) mycelia were transferred and incubated in 50 mL MM (pH 4.5) with 50 mm GA for 55 h. Additionally, 30 g (wet weight) mycelia of SDP20.6 (Δ*gaaC*) were transferred and incubated in 1 L MM (pH 4.5) with 50 mm GA for 55 h. For RNA‐seq analysis, pregrown mycelia were washed with MM with no carbon source (pH 6) and 2.5 g (wet weight) were transferred to 50 mL MM (pH 6) with 25 mm GA and grown for 2 h. All incubations were carried out in a rotary shaker at 30 °C and 250 r.p.m.

### Construction of gene deletion strains

Protoplast‐mediated transformation of *A. niger*, purification of the transformants and genomic DNA extraction were performed as described 36.

To construct the deletion cassettes, 5′ and 3′ flanks of the *gaaA*,* gaaB*,* gaaC*, and *gaaD* genes were PCR‐amplified using the primer pairs listed in Table S2 with N402 genomic DNA as template. For all cloning experiments *Escherichia coli* strain DH5α was used. To create SDP22.1 (Δ*gaaA*), SDP21.5 (Δ*gaaB*), and SDP20.6 (Δ*gaaC*), gene deletion cassettes were made using MultiSite Gateway Three‐fragment Vector Construction Kit (Invitrogen, Carlsbad, CA, USA) according to the supplier's instructions. *Aspergillus oryzae pyrG* gene flanked by AttB1 and AttB2 sites was amplified by PCR using the primer pair listed in Table S2 and plasmid pMA172 37 as template. *gaaA*,* gaaB*, and *gaaC* deletion cassettes containing 5′ and 3′ flanks of the target genes with *A. oryzae pyrG* gene in between were obtained by restriction digestion. To create EA1.1 (Δ*gaaD*), 5′ flank of *gaaD* was ligated into pJET1.2/blunt cloning vector (Thermo Fisher Scientific, Carlsbad, CA, USA) and amplified in *E. coli*. Following plasmid isolation, the 5′ flank was excised using restriction enzymes *Kpn*I and *Xho*I, ligated into *Kpn*I‐*Xho*I opened pBluescript II SK(+) (Agilent Technologies, La Jolla, CA, USA) and amplified in *E. coli*. *Aspergillus oryzae pyrG* gene was obtained from plasmid pMA172 37 by restriction digestion with *Hind*III and *Xho*I. Isolated pBluescript II SK(+) plasmid containing the 5′ flank was opened with restriction enzymes *Xho*I and *Not*I, and the *A. oryzae pyrG* gene as *Xho*I‐*Not*I fragment and *Hind*III‐*Not*I fragment of the *gaaD* 3′ flank were ligated into the plasmid. Ligation product was amplified in *E. coli* and the linear deletion cassette was obtained by PCR amplification from the plasmid using primers gaaDP1‐*Kpn*I and gaaDP4‐NotI. Deletion cassettes were introduced into the *pyrG* ^*−*^ strain N593.20. Gene deletions were confirmed *via* southern blot analysis.

### Gene expression analysis

Northern blot and RNA‐seq analyses were performed as described 19 with minor modifications: For northern blot analysis, total RNA was extracted using TRIzol reagent (Life Technologies, Carlsbad, CA, USA). Probes were PCR‐amplified using the N402 genomic DNA and the primer pairs listed in Table S2.

### Chemical analysis

One milliliter culture samples were taken 7, 24, 31, 48, and 55 h after the transfer of mycelia to MM with GA. About 250 μL of each culture sample was centrifuged at 16 000 ***g*** for 30 min and the supernatant was transferred to a new microfuge tube. After adding 1× volume of cold methanol (−20 °C), the sample was incubated on ice for 15 min and centrifuged at 16 000 ***g*** for 30 min. The supernatant was collected in a new microfuge tube and 1× volume of 0.1% formic acid was added. Metabolites in the extracellular culture fluids were analyzed by high pressure liquid chromatography–high‐resolution mass spectrometry. Aliquots were loaded, using a Series 200 micropump (PerkinElmer), onto a reversed‐phase Eclipse C18 2.1 × 150 mm column (Agilent, Santa Clara, CA, USA) connected in‐line to a 7 Tesla LTQ‐FT‐ICR mass spectrometer (Thermo Electron Corporation, San Jose, CA, USA) and negative mode electrospray ionization spectra were acquired at a resolution of 100 000 at 200 *m*/*z*. Absolute GA concentration was calculated using a standard dilution calibration curve of commercially obtained GA (Chemodex). Standards for l‐galactonate and 2‐keto‐3‐deoxy‐l‐galactonate were not available, therefore, these metabolites were assigned based on accurate mass alone (matched within a 5 p.p.m. *m*/*z* window) and relative amounts in terms of extracted ion chromatograms peak areas were compared. One liter culture of SDP20.6 (Δ*gaaC*) was filtered through sterile myracloth 55 h after the transfer of mycelia to MM with GA, and the filtrate was stored at −80 °C. After freeze‐drying, dry materials from SDP20.6 (*ΔgaaC*) extracellular culture fluid were dissolved in D_2_O (Sigma Aldrich) for structural investigation by Nuclear Magnetic Resonance Spectroscopy (NMR). Spectra were recorded with a Varian VNMRS‐500 MHz at 25 °C. The presence of 2‐keto‐3‐deoxy‐l‐galactonate was confirmed by ^1^H‐NMR and ^13^C‐NMR.

### Bioinformatics

RNA‐seq data were analyzed as described previously 19. Differential expression was identified by Student's *t*‐test with a *P*‐value cut‐off of 0.05. RNA‐seq data for FP‐1132.1 (reference strain) and SDP20.6 (*ΔgaaC*) were submitted to Gene Expression Omnibus 38 with accession numbers GSE80227 19 and GSE95776 (this study), respectively.

## Results

### Growth analysis of d‐galacturonic acid catabolic pathway deletion mutants

*Aspergillus niger* GA catabolic pathway deletion mutants, *∆gaaA*,* ∆gaaB*,* ∆gaaC*,* and ∆gaaD*, were constructed and were verified by southern blot analysis (Fig. S1). We compared the growth phenotype of the strains on monomeric and polymeric carbon sources (Fig. 1, Fig. S2). Disruption of *gaaA* and *gaaD* resulted in reduced growth and sporulation on plates containing GA or PGA as carbon source. However, both mutants showed better growth on plates containing MM with GA compared to plates containing MM with no carbon source, indicating that they can still metabolize GA. The *∆gaaB* and *∆gaaC* mutants showed a more drastically reduced growth on plates containing GA, PGA, or AP (Fig. 1B). The growth defects of the GA catabolic pathway deletion mutants on GA plates were confirmed in microtiter plate‐based growth assays (Fig. 1C, Fig. S2A). None of the GA catabolic pathway deletion mutants exhibited defects in growth on other carbon sources tested, except that the deletion of *gaaD*, also known as the l‐arabinose reductase gene *larA*, resulted in a poor growth on l‐arabinose (Fig. S2B), confirming previous observations 15. The inability of *∆gaaB* or *∆gaaC* to use GA as a carbon source suggests that there are no functionally redundant enzymes capable of replacing GaaB and GaaC.

### *∆gaaB* and *∆gaaC* accumulate the d‐galacturonic acid catabolic pathway intermediates l‐galactonate and 2‐keto‐3‐deoxy‐l‐galactonate, respectively

**Figure 2:**
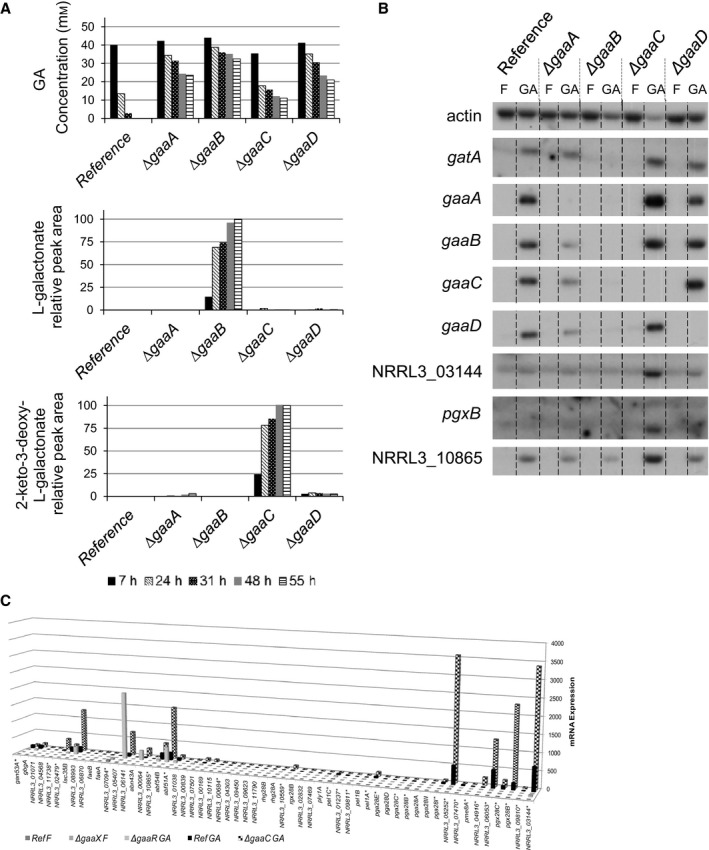
Metabolic and gene expression analyses of *Aspergillus niger* GA catabolic pathway deletion mutants *∆gaaA*,* ∆gaaB*,* ∆gaaC, and ∆gaaD* (A) Extracellular GA, l‐galactonate, and 2‐keto‐3‐deoxy‐l‐galactonate concentration in cultures of the reference strain (FP‐1132.1) and GA catabolic pathway deletion mutants. GA concentration is given in mm and l‐galactonate and 2‐keto‐3‐deoxy‐l‐galactonate amounts are presented as ion chromatogram peak areas relative to *∆gaaB* 55 h and *∆gaaC* 55 h samples, respectively. (B) Northern blot analysis of selected GA‐responsive genes in the reference strain (MA249.1) and GA catabolic pathway deletion mutants. Actin (NRRL3_03617) was used as a control. (C) RNA‐seq analysis of pectinase genes in the reference strain (FP‐1132.1) and *∆gaaC* in GA (FPKM). Expression in *∆gaaR* in GA (FPKM) 19 and in the reference strain (MA234.1) and *∆gaaX* in d‐fructose (TPM) 20 was shown for comparison. Pectinase genes that belong to the GaaR/GaaX panregulon 20 are indicated with an asterisk. Strains were pregrown in CM with 2% d‐fructose. For metabolic analysis, mycelia were transferred to and grown in MM containing 50 mm GA. For northern blot analysis, mycelia were transferred to and grown in MM containing 50 mm d‐fructose (F) or GA for 2 h. For RNA‐seq analysis, mycelia were transferred to and grown in MM containing 25 mm GA for 2 h.

Since the roles of GaaB and GaaC in GA catabolism cannot be replaced by redundant enzymes, we expect the accumulation in the medium of the corresponding enzyme substrate in *∆gaaB* and *∆gaaC*, as shown previously 7, 8. The extracellular GA concentration and the extracellular metabolites were examined by FT‐ICR mass spectrometry over time during growth in GA. This analysis revealed that the reference strain utilized all GA in the medium within 48 h of growth, whereas in the GA catabolic pathway deletion mutants GA was still present in the medium after 55 h of growth (Fig. 2A). In *∆gaaA* and *∆gaaD*, the concentration of GA gradually decreased to approximately 45% of the initial GA concentration in the medium, which reflects the slow catabolism of GA in these mutants. *∆gaaB* consumed about 35% of the initial GA in 55 h and secreted l‐galactonate. The time course consumption of GA by *∆gaaB* was proportional to its release of l‐galactonate (Fig. 2A). The *∆gaaC* mutant took up about 78% of the initial GA in 55 h, and extracellular 2‐keto‐3‐deoxy‐l‐galaconate accumulated in the medium of the *∆gaaC* mutant over time (Fig. 2A). The presence of 2‐keto‐3‐deoxy‐l‐galactonate in the extracellular culture fluid of the *∆gaaC* mutant was confirmed by structural resolution by ^1^H‐NMR and ^13^C‐NMR (Fig. S3).

### Expression of d‐galacturonic acid‐responsive genes is increased in *∆gaaC*

Genes involved in the degradation of the pectic substructures PGA (e.g., NRRL3_03144 exo‐polygalacturonase and *pgx28B*) and rhamnogalacturonan I (RG‐I) (e.g., NRRL3_10865 alpha‐*N*‐arabinofuranosidase), GA transport (*gatA*), and GA catabolism (*gaaA‐D*) have been shown to be induced in the presence of GA 5, 18 and are part of the proposed GaaR/GaaX‐controlled gene regulon 20. To test the effect of the GA catabolic pathway gene deletions on the induction of GA‐responsive genes, northern blot analysis was performed. The reference and *∆gaaA*,* ∆gaaB*,* ∆gaaC* and *∆gaaD* strains were pregrown in d‐fructose medium and transferred to either GA or d‐fructose medium. Rapid induction of *gatA*,* gaaA*,* gaaB*,* gaaC*,* gaaD*, and NRRL3_10865 was observed in the reference strain upon transfer from d‐fructose to GA as expected (Fig. 2B). Induction of these genes upon transfer to GA was also found in *∆gaaA*, but at lower levels compared to the reference strain. The induction of GA‐responsive genes was nearly absent in *∆gaaB*. As shown in Fig. 2B, deletion of *gaaC* resulted in a hyperinduction of GA‐responsive genes, especially pectinases (NRRL3_03144, *pgx28B*, and NRRL3_10865). Expression of *gatA*,* gaaA*,* gaaB*,* gaaC*, and the pectinases in *∆gaaD* was similar to the expression in the reference strain (Fig. 2B).

### Transcriptome analysis of *ΔgaaC*

In order to analyze the expression of a larger number of genes controlled by GaaR/GaaX activator–repressor module in *∆gaaC*, a genome‐wide gene expression analysis was performed using RNA‐seq. The reference strain and the *∆gaaC* mutant were pregrown in d‐fructose medium and transferred to GA medium. Seventeen of the 53 GaaR/GaaX panregulon genes were significantly upregulated (FC ≥ 2 and *P*‐value ≤ 0.05) in the *ΔgaaC* mutant cultured in GA as compared to the reference strain (Table 1, Table S3). These 17 genes include *gaaA* and 6 pectinases (NRRL3_03144, *pgx28B*, NRRL3_05252, NRRL3_04916, NRRL3_10559, and NRRL3_11738), as well as genes encoding four transporters and six genes for which the function has not yet been established. The expression of 24 of the remaining GaaR/GaaX panregulon genes was higher in *ΔgaaC* compared to the reference strain, but differences were relatively small and did not pass the stringent *P*‐value of ≤ 0.05.

**Table 1:** RNA‐seq analysis of 53 genes of the GaaR‐GaaX panregulon 20 in *ΔgaaC* in GA. 27 genes belonging to GaaR‐GaaX core regulon 20 are written in bold. Expression values (FPKM) are averages of duplicates. Significantly upregulated genes (FC ≥ 2 and *P*‐value ≤ 0.05) are highlighted

Gene ID NRRL3	Gene ID CBS513.88	Description^a^	Gene name	Ref	*ΔgaaC*	FC *ΔgaaC*/Ref	*P*‐value
**NRRL3_00958**	**An14g04280**	**d‐** **galacturonic acid transporter GatA**	***gatA***	888.35	1062.68	1.20	6.95E‐02
**NRRL3_03144**	**An12g07500**	**Exo‐polygalacturonase**		698.90	3384.63	4.84	1.34E‐02
**NRRL3_05260**	**An02g12450**	**Exo‐polygalacturonase Pgx28C**	***pgx28C***	99.93	192.85	1.93	9.11E‐02
**NRRL3_05649**	**An02g07720**	**2‐Keto‐3‐deoxy‐** **l‐** **galactonate aldolase GaaC**	***gaaC***	5658.32	14.60	0.00	2.88E‐04
**NRRL3_05650**	**An02g07710**	**d** **‐Galacturonic acid reductase GaaA**	***gaaA***	2599.98	6710.72	2.58	1.04E‐02
**NRRL3_06053**	**An02g02540**	**Carbohydrate esterase family 16 protein**		522.81	1301.08	2.49	8.01E‐02
**NRRL3_06890**	**An16g05390**	**l** **‐Galactonate dehydratase GaaB**	***gaaB***	11309.00	13990.90	1.24	1.91E‐01
**NRRL3_08281**	**An03g06740**	**Exo‐polygalacturonase Pgx28B**	***pgx28B***	200.31	2306.06	11.51	2.82E‐02
**NRRL3_08663**	**An03g01620**	**MFS‐type sugar/inositol transporter**		106.09	227.29	2.14	1.71E‐01
**NRRL3_10050**	**An11g01120**	**l** **‐Glyceraldehyde reductase GaaD**	***gaaD***	8104.43	7499.78	0.93	5.79E‐01
**NRRL3_10865**	**An08g01710**	**Alpha‐** ***N*** **‐arabinofuranosidase**		201.62	440.98	2.19	1.92E‐01
**NRRL3_01237**	**An19g00270**	**Pectin lyase**		18.95	3.68	0.19	9.55E‐03
**NRRL3_02479**	**An01g10350**	**Exo‐beta‐1,4‐galactanase**		137.63	170.01	1.24	5.21E‐01
**NRRL3_05252**	**An02g12505**	**Pectin methylesterase**		558.37	3569.08	6.39	2.07E‐02
**NRRL3_07470**	**An04g09690**	**Pectin methylesterase**		30.16	12.81	0.42	4.22E‐02
**NRRL3_08325**	**An03g06310**	**Pectin methylesterase Pme8A**	***pme8A***	6.54	6.74	1.03	8.79E‐01
**NRRL3_10559**	**An18g04810**	**Glycoside hydrolase family 28 protein**		20.00	97.18	4.86	1.19E‐02
NRRL3_00965	An14g04370	Pectin lyase Pel1A	*pel1A*	56.54	113.40	2.01	3.58E‐01
NRRL3_04281	An07g00780	MFS‐type transporter		90.41	106.00	1.17	5.05E‐01
NRRL3_09810	An11g04040	Exo‐polygalacturonase		10.65	35.99	3.38	7.58E‐02
**NRRL3_08194**	**An04g00790**	**Repressor of ** **d‐** **galacturonic acid utilization**	***gaaX***	381.34	529.21	1.39	1.97E‐01
NRRL3_00684	An14g01130	Rhamnogalacturonan lyase		5.77	13.23	2.29	2.61E‐01
NRRL3_01606	An01g00330	Alpha‐*N*‐arabinofuranosidase Abf51A	*abf51A*	87.96	111.63	1.27	4.97E‐01
NRRL3_02571	An01g11520	Endo‐polygalacturonase Pga28I	*pga28I*	56.38	59.67	1.06	5.83E‐01
NRRL3_02835	An01g14670	Endo‐polygalacturonase Pga28E	*pga28E*	4.26	13.51	3.17	9.99E‐02
NRRL3_04153	An15g07160	Pectin lyase		35.48	19.78	0.56	3.56E‐02
NRRL3_04916	An07g08940	Carbohydrate esterase family 16 protein		13.41	221.16	16.49	4.37E‐02
NRRL3_05859	An02g04900	Endo‐polygalacturonase Pga28B	*pga28B*	15.10	4.12	0.27	9.36E‐02
NRRL3_07094	An16g02730	Endo‐1,5‐alpha‐arabinanase		4.57	3.48	0.76	2.43E‐01
NRRL3_08805	An05g02440	Endo‐polygalacturonase Pga28C	*pga28C*	5.26	7.27	1.38	1.85E‐01
NRRL3_09811	An11g04030	Pectin lyase		0.51	0.11	0.21	6.88E‐02
NRRL3_10643	An18g05940	Arabinogalactanase Gan53A	*gan53A*	105.64	67.21	0.64	2.70E‐01
NRRL3_11738	An06g00290	Beta‐galactosidase		28.91	319.96	11.07	4.60E‐02
NRRL3_00502	An09g06200	Hypothetical protein		14.07	41.41	2.94	1.16E‐01
**NRRL3_00660**	**An14g00860**	**Carboxylesterase**		74.22	825.36	11.12	4.58E‐02
NRRL3_00957	An14g04260	B3/B4 domain‐containing protein		7.87	13.03	1.66	2.87E‐01
NRRL3_01073	An14g05840	O‐methyltransferase, COMT‐type		3.22	11.45	3.55	1.39E‐02
**NRRL3_01127**	**An14g06500**	**Dihydroxyacetone kinase**		584.25	203.94	0.35	1.55E‐02
**NRRL3_01398**	**An13g02090**	**MFS‐type transporter**		26.10	96.31	3.69	1.69E‐02
NRRL3_02770	An01g13880	MFS‐type transporter		3.71	6.43	1.73	9.57E‐02
NRRL3_03291	An12g05600	Heterokaryon incompatibility protein		0.80	6.04	7.60	6.39E‐02
NRRL3_03292	An12g05590	Carboxylesterase		0.25	1.72	6.88	3.30E‐01
**NRRL3_03342**	**An12g04990**	**Short‐chain dehydrogenase/reductase**		151.58	706.28	4.66	1.05E‐02
NRRL3_03467	An12g03550	MFS‐type transporter		4.91	92.55	18.85	2.61E‐02
**NRRL3_06244**	**An02g00140**	**Glycoside hydrolase family 43 protein**		80.90	137.44	1.70	1.81E‐01
NRRL3_07382	An16g00540	Alpha‐l‐fucosidase		2.29	8.06	3.53	4.41E‐02
NRRL3_08499	An03g03960	Uncharacterized protein		13.64	45.86	3.36	6.05E‐03
**NRRL3_08833**	**n.a.**	**Hypothetical protein**		4.29	1.87	0.44	2.27E‐02
NRRL3_09862	An11g03510	Hypothetical protein		0.43	0.20	0.45	5.62E‐01
**NRRL3_09863**	**An11g03500**	**Alpha‐hydroxy acid dehydrogenase, FMN‐dependent**		59.53	64.98	1.09	2.85E‐01
**NRRL3_10558**	**An18g04800**	**Alpha‐** **l‐** **rhamnosidase**		17.04	109.06	6.40	3.54E‐02
**NRRL3_11054**	**An08g04040**	**MFS‐type sugar/inositol transporter**		693.37	4713.62	6.80	8.89E‐03
NRRL3_11710	An06g00620	MFS‐type sugar/inositol transporter		341.35	1977.10	5.79	2.76E‐02

a Descriptions were obtained from manual annotation (manuscript in preparation).

In addition to GaaR/GaaX‐controlled genes, we also compared the expression of all 58 pectinases identified in the genome of *A. niger* 2 between the reference strain and the *ΔgaaC* mutant (Table S4, Fig. 2C). Apart from the six pectinases that depend on GaaR for induction 19, nine additional pectinases acting on the RG‐I backbone and arabinan and arabinogalactan side chains were significantly upregulated (FC ≥ 2 and *P*‐value ≤ 0.05) in the *ΔgaaC* mutant compared to the reference strain (Table 2). It has been reported that many of these genes are regulated by transcription factors RhaR (NRRL3_02832, NRRL3_07501, NRRL3_07501, and *faeB*), XlnR (NRRL3_05407 and *lac35B*), or AraR (*lac35B*), which are required for the utilization of l‐rhamnose, xylan/d‐xylose, and arabinan/l‐arabinose, respectively 39, 40, 41, 42. To address the possibility that deletion of *gaaC* affected the expression of these genes *via* their specific transcription factors, the expression of *rhaR*,* xlnR*, and *araR* was analyzed in more detail. Expression of *rhaR* (FC = 5.84 and *P*‐value = 4.76E‐03) and *xlnR* (FC = 2.68 and *P*‐value = 5.60E‐03) was significantly higher in *ΔgaaC*, which might explain the upregulation observed in these genes. The *araR* gene was not significantly differentially regulated in the *ΔgaaC* mutant.

**Table 2:** RNA‐seq analysis of nine pectinase genes that were significantly upregulated in *ΔgaaC* in GA and do not belong to the GaaR‐GaaX panregulon 20

Gene ID NRRL3	Gene ID CBS513.88	Description^a^	Gene name	Ref	*ΔgaaC*	FC *ΔgaaC*/Ref	*P*‐value
NRRL3_02832	An01g14650	Glycoside hydrolase family 28 protein		1.49	12.95	8.69	1.21E‐02
NRRL3_09450	An11g08700	Endo‐rhamnogalacturonase		1.75	4.34	2.48	3.39E‐02
NRRL3_07501	An04g09360	Carbohydrate esterase family 12 protein		17.42	87.29	5.01	4.60E‐02
NRRL3_00839	An14g02920	Glycoside hydrolase family 105 protein		3.61	22.81	6.32	5.98E‐03
NRRL3_05407	An02g10550	Endo‐1,5‐alpha‐arabinanase		103.20	702.79	6.81	1.45E‐02
NRRL3_02931	An12g10390	Feruloyl esterase FaeB	*faeB*	4.17	16.38	3.93	3.08E‐02
NRRL3_02630	An01g12150	Beta‐galactosidase Lac35B	*Lac35B*	172.89	1259.38	7.28	3.28E‐02
NRRL3_04568	An07g04420	Exo‐beta‐1,4‐galactanase		0.23	9.58	41.63	7.17E‐03
NRRL3_01071	An14g05820	Beta‐galactosidase		0.75	8.06	10.74	2.90E‐02

a Descriptions were obtained from manual annotation (manuscript in preparation).

## Discussion

In this study, we used GA catabolic pathway deletion mutants to investigate the induction mechanism of the GA‐responsive genes in *A. niger*. We observed that the *gaaA* and the *gaaD* deletion mutants show reduced growth on GA or PGA compared to the reference strain, whereas growth of *∆gaaB* and *∆gaaC* is more severely reduced on GA, PGA, or AP (Fig. 1B,C). These results are in line with the previous reports showing the inability of *∆gaaB* and *∆gaaC* to grow on GA 7, 8. *ΔgaaA* was reported to be unable to grow on GA in a previous study 6, where the tenuous growth of *ΔgaaA* could have been interpreted as no growth. GA catabolic pathway deletion mutants derived from N593.20 in this study and from ATCC1015 in previous studies 6, 7, 8 showed the same growth defects on GA (unpublished results), excluding the possibility of a phenotypic difference caused by strain background.

Deletion of *gaaB* and *gaaC* severely impaired growth on MM containing GA (Fig. 1B,C), indicating that there are no alternative enzymes replacing GaaB and GaaC. The residual growth of *∆gaaA* and *∆gaaD* on GA indicates that GA is catabolized in these reductase deletion mutants *via* partially redundant enzymes. In *B. cinerea*, there are two nonhomologous d‐galacturonate reductases, BcGar1, and BcGar2. While single gene deletion mutants (*∆Bcgar1* or *∆Bcgar2)* could still grow on GA, the double gene deletion mutant *∆Bcgar1∆Bcgar2* showed a complete loss of growth 9. *Aspergillus niger* also contains a BcGar1 ortholog, NRRL3_06930, which shows no protein homology to GaaA. As in *B. cinerea*, NRRL3_06930 might enable the residual growth of *∆gaaA* on GA. However, the expression of NRRL3_06930 is considerably lower than the expression of *gaaA* in GA, and unlike the expression of *gaaA*, does not depend on GaaR or GaaX 19, 20. It is also possible that the two dehydrogenases belonging to the GaaR/GaaX panregulon, NRRL3_03342, and NRRL3_09863, partially replace GaaA or GaaD.

The recently proposed model related to the regulation of GA‐responsive gene expression 20 postulates that under noninducing conditions the repressor GaaX inhibits the transcriptional activity of GaaR. The repressing activity of GaaX is suggested to be lost in the presence of an inducer and subsequent activation of GaaR, resulting in the induction of GA‐responsive genes in *A. niger* 20. The results of metabolic and northern blot analyses indicate that accumulation of 2‐keto‐3‐deoxy‐l‐galactonate in *∆gaaC* is responsible for the induction of the GA‐responsive genes. In other words, the pathway intermediate 2‐keto‐3‐deoxy‐l‐galactonate, and not GA or l‐galactonate, is the physiological inducer of the GA‐responsive genes in *A. niger* (Fig. 2A,B). In the *ΔgaaA* mutant, we postulate that GA is converted into l‐galactonate *via* partially redundant enzymes (see above) and the 2‐keto‐3‐deoxy‐l‐galactonate produced is enough for the induction of GA‐responsive genes. However, this induction is lower compared to the reference strain (Fig. 2B). This result is supported by a previous finding that *gaaB* and *gaaC* were expressed at lower levels in *ΔgaaA* compared to the reference strain 6. In contrast, *ΔgaaB* possibly does not produce 2‐keto‐3‐deoxy‐l‐galactonate from l‐galactonate, since the growth phenotype of the *ΔgaaB* mutant suggests that there are no functionally redundant enzymes replacing GaaB. As a result, expression of GA‐responsive genes is not induced in *ΔgaaB* (Fig. 2B). Reduced expression of *gatA*,* gaaA*, and *gaaC* in the *ΔgaaB* mutant was also observed previously 7.

RNA‐seq analysis of *ΔgaaC* revealed significant upregulation of several genes from the GaaR/GaaX panregulon involved in pectin breakdown and GA utilization, as well as genes with currently unknown link to GA utilization, such as transporters that might facilitate the faster GA transport in *∆gaaC* compared to other GA catabolic pathway deletion mutants observed both in this study (Fig. 2A) and previous studies 6, 7, 8. Deletion of *gaaC* also induced the expression of several pectinases acting on RG‐I that do not belong to GaaR/GaaX panregulon (Table 2). A possible explanation is that starvation in *ΔgaaC* results in the induction of these genes. Several pectinases acting on side chains of RG‐I, including NRRL3_05407, *lac35B* and NRRL3_07501, were previously reported to be induced upon starvation 43. Another explanation is that the increased transcript levels of *rhaR* and *xlnR* results in an increase in the expression of these genes that were suggested to be under control of RhaR and XlnR (see above).

Although both *∆gaaB* and *∆gaaC* cannot utilize GA, residual growth of *∆gaaC* was observed on AP, whereas the growth of *∆gaaB* on AP was more impaired (Fig. 1B). This could be explained by the high capacity of *∆gaaC* to secrete pectinases acting on RG‐I and release monosaccharides (l‐arabinose, l‐rhamnose, d‐galactose) other than GA to support growth, and the less efficient pectinase production in *∆gaaB*.

Previously, we identified 53 genes as the GaaR/GaaX panregulon downregulated in *ΔgaaR* under inducing condition and/or upregulated in *ΔgaaX* under non‐inducing condition. However, only a core set of 27 genes was significantly differentially regulated under both conditions 19, 20, and only 17 of 53 panregulon genes, 10 of which belong to the core regulon, were hyperinduced in response to deletion of *gaaC* (Table 1), demonstrating the complex regulation of GA‐responsive gene expression. A dynamic equilibrium is suggested to exist between the free and DNA‐bound states of a transcription factor, and the binding of a transcription factor to the promoters of its target genes depends on its concentration, as well as its cooperative/competitive interactions with other proteins and the chromatin accessibility 44, 45. Deletion of *gaaR* would result in the lack of GaaR in the cell, whereas deletion of *gaaX* or intracellular accumulation of 2‐keto‐3‐deoxy‐l‐galactonate in *∆gaaC* would, possibly to different degrees, increase the concentration of active GaaR by elimination or reducing the repressing activity of GaaX. GaaR concentration might also be regulated transcriptionally: *gaaX* is highly upregulated in GA 5, whereas *gaaR* expression is significantly increased in the *∆gaaC* mutant (FC = 5.10 and *P*‐value = 7.88E‐03). Moreover, different levels of CreA mediated repression on different GA‐responsive genes 18 and accessibility of the promoter regions of these genes under different conditions might play a role in the observed differences in gene regulation. Condition specific cross‐regulation between transcription factors and coregulation of target genes might add additional complexity to GA‐responsive gene expression, as discussed above.

To conclude, in this study we identified the GA catabolic pathway intermediate 2‐keto‐3‐deoxy‐l‐galactonate as the probable inducer of the GA‐responsive genes in *A. niger*. Considering that both the GA catabolic pathway enzymes and the GaaR/GaaX activator–repressor module is evolutionarily conserved in the Pezizomycotina subdivision of Ascomycetes 5, 20, it is highly probable that the mechanism by which 2‐keto‐3‐deoxy‐l‐galactonate acts as an inducer and interacts with the activator–repressor module is also conserved.

## Author contributions

EA, CK, TGH, SdP, MA, MDF, TTMP performed experiments. EA, MDF, MP, MVAP performed bioinformatics analysis. EA, JV, AT, RPdV, and AFJR wrote the manuscript with input of all authors.

## Supporting information


**Fig. S1.** Verification of the GA catabolic pathway deletion strains (A) *∆gaaA* (SDP22.1), (B) *∆gaaB* (SDP21.5), (C) *∆gaaC* (SDP20.6), *and* (D) *∆gaaD* (EA1.1) *via* southern blot analysis of genomic DNA.
**Fig. S2.** Growth profile of the *Aspergillus niger* reference strain (MA249.1) and GA catabolic pathway deletion mutants *∆gaaA*,* ∆gaaB*,* ∆gaaC, and ∆gaaD*.
**Fig. S3.** (A) Predominant form (pyranose) of 2‐keto‐3‐deoxy‐l‐galactonate in the extracellular culture fluid of *Aspergillus niger ∆gaaC* grown in MM containing 50 mm GA for 55 h.

**Table S1.** Strains used in this study.

**Table S2.** Primers used in this study.

**Table S3.** RNA‐seq analysis of 53 genes of the GaaR‐GaaX panregulon [20] in *∆gaaC* and *ΔgaaR* in GA and in *ΔgaaX* in d‐fructose.

**Table S4.** RNA‐seq analysis of pectinases in *∆gaaC* and *∆gaaR* in GA and in *∆gaaX* in d‐fructose.
