# Supplementary material for: The pathway intermediate 2‐keto‐3‐deoxy‐L‐galactonate mediates the induction of genes involved in D‐galacturonic acid utilization in Aspergillus niger
Source: FEBS Lett. 2017 May 6;591(10):1408–18. doi: 10.1002/1873-3468.12654 (PMC5488244; doi:10.1002/1873-3468.12654)
Supplement: Supplementary file 1 — Fig. S1. Verification of the GA catabolic pathway deletion strains (A) ∆gaaA (SDP22.1), (B) ∆gaaB (SDP21.5), (C) ∆gaaC (SDP20.6), and (D) ∆gaaD (EA1.1) via southern blot analysis of genomic DNA. Fig. S2. Growth profile of the Aspergillus niger reference strain (MA249.1) and GA catabolic pathway deletion mutants ∆gaaA, ∆gaaB, ∆gaaC, and ∆gaaD. Fig. S3. (A) Predominant form (pyranose) of 2‐keto‐3‐deoxy‐l‐galactonate in the extracellular culture fluid of Aspergillus niger ∆gaaC grown in MM containing 50 mm GA for 55 h. [file FEB2-591-1408-s001.docx]

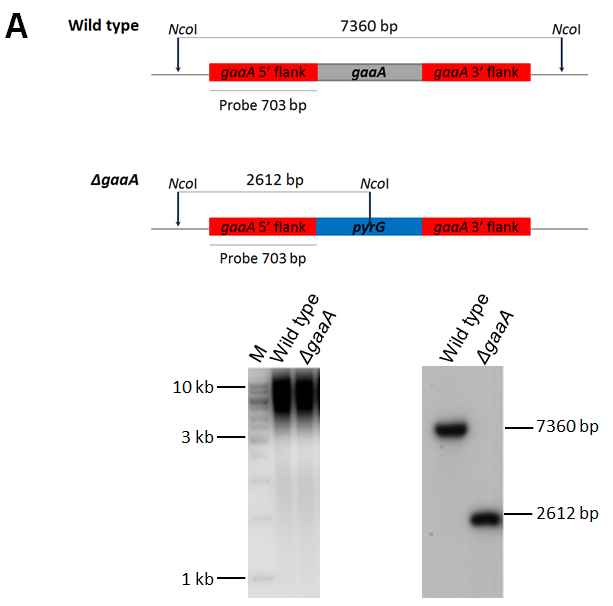

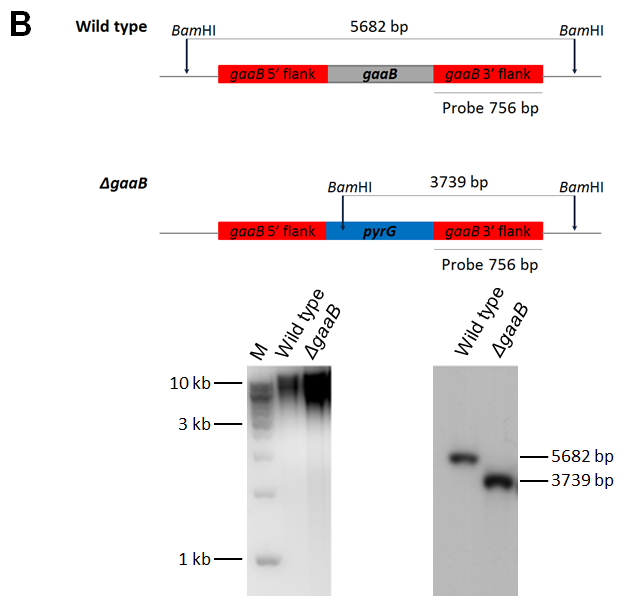


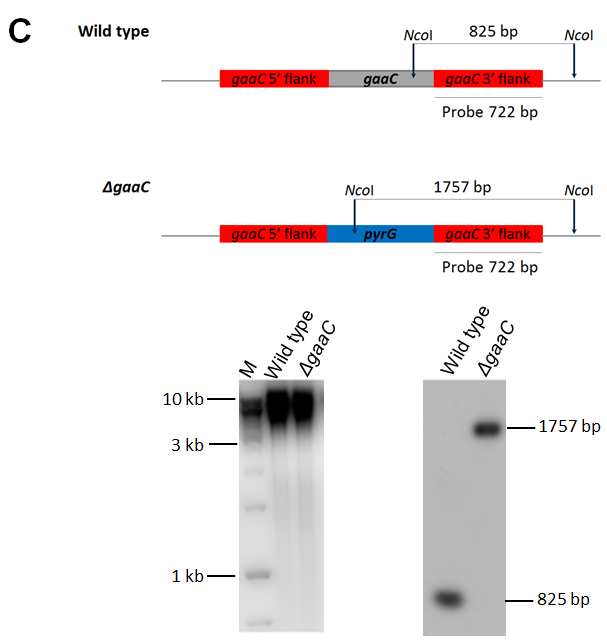

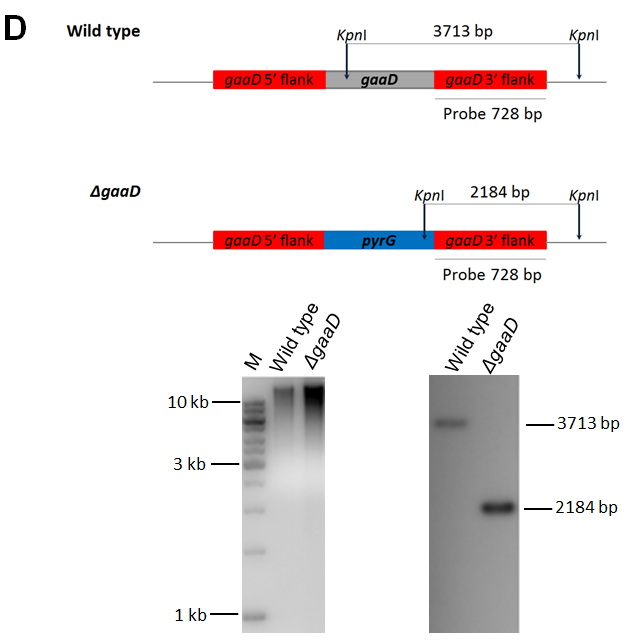


**Figure S1** Verification of the GA catabolic pathway deletion strains (A) *∆gaaA* (SDP22.1), (B) *∆gaaB* (SDP21.5), (C) *∆gaaC* (SDP20.6) *and* (D) *∆gaaD* (EA1.1) via Southern blot analysis of genomic DNA. For each gene deletion, schematic representation of the target gene locus in the wild type (N593.20) and gene deletion strain are shown together with the predicted sizes of the DNA fragments hybridizing with the indicated probes. Left panel: agarose gel stained with ethidium bromide. Marker size (M, in kb) is indicated. Right panel: Southern blot after hybridization.

**A**

**
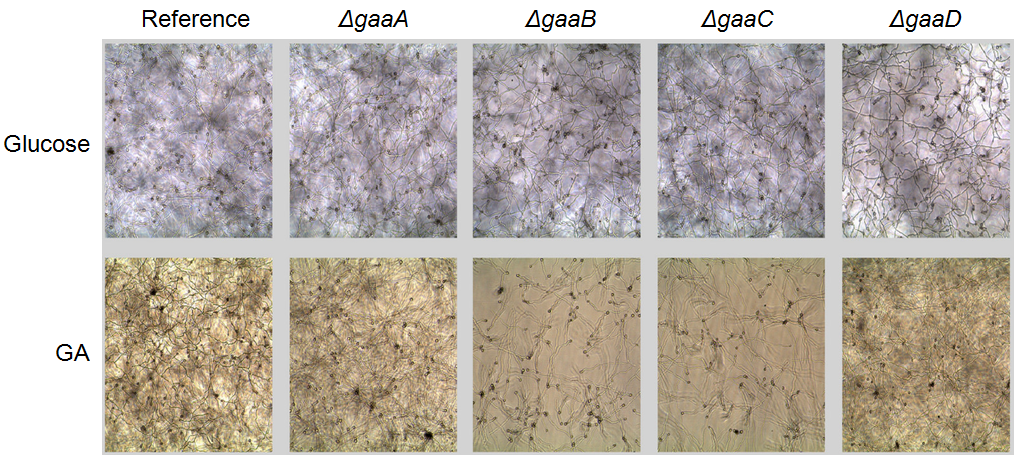
**

**B**


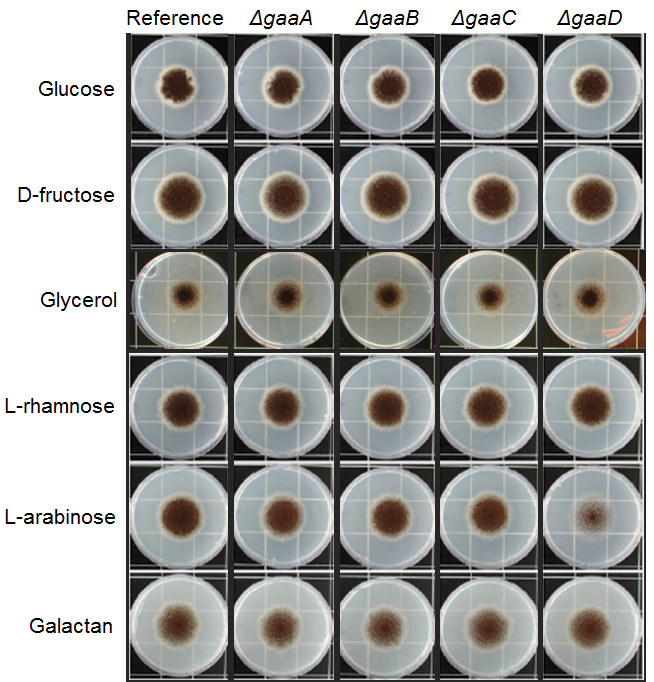


**Figure S2** Growth profile of the *A. niger* reference strain (MA249.1) and GA catabolic pathway deletion mutants *∆gaaA*, *∆gaaB*, *∆gaaC and* *∆gaaD*. Strains were grown (A) in microtiter plate in liquid medium with 50 mM glucose or GA for 69 h at 30 °C, and (B) on solid MM with 50 mM monomeric or 1% polymeric carbon sources for 7 days at 30 °C. Microscopic pictures of cultures in microtiter plate were taken with a Leica ICC50 inverted microscope.

**A**

**B**


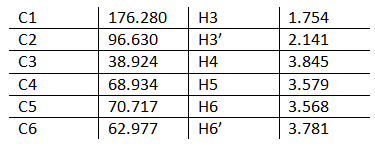


**Figure S3** (A) Predominant form (pyranose) of 2-keto-3-deoxy-L-galactonate in the extracellular culture fluid of *A. niger ∆gaaC* grown in MM containing 50 mM GA for 55 h. R1 and R2 refer to carbonyl group and hydroxyl group, respectively. (B) ^1^H and ^13^C chemical shifts (ppm) of 2-keto-3-deoxy-L-galactonate, consistent with data published previously [Kuorelahti *et al.*, 2006].
