# Supplementary material for: The pathway intermediate 2‐keto‐3‐deoxy‐L‐galactonate mediates the induction of genes involved in D‐galacturonic acid utilization in Aspergillus niger
Source: FEBS Lett. 2017 May 6;591(10):1408–18. doi: 10.1002/1873-3468.12654 (PMC5488244; doi:10.1002/1873-3468.12654)
Supplement: Supplementary file 2 — Table S1. Strains used in this study. [file FEB2-591-1408-s002.docx]

**Table S1** Strains used in this study

| **Strain** | **Genotype** | **Description** | **Reference** |
| --- | --- | --- | --- |
| N593.20 | *cspA1, pyrG^-^, kusA::amdS* | *ΔkusA* in N593 | Alazi *et al.*, 2016 |
| FP-1132.1 | *cspA1, pyrG^-^::AOpyrG, kusA::amdS* | Restored *pyrG* in N593.20 | Alazi *et al.*, 2016 |
| MA249.1 | *cspA1, pyrG^-^::ANpyrG, kusA::amdS* | Restored *pyrG* in N593.20 | This study |
| SDP22.1 | *cspA1, pyrG^-^, kusA::amdS, gaaA::AOpyrG* | *ΔgaaA* in N593.20 | This study |
| SDP21.5 | *cspA1, pyrG^-^, kusA::amdS, gaaB::AOpyrG* | *ΔgaaB* in N593.20 | This study |
| SDP20.6 | *cspA1, pyrG^-^, kusA::amdS, gaaC::AOpyrG* | *ΔgaaC* in N593.20 | This study |
| EA1.1 | *cspA1, pyrG^-^, kusA::amdS, gaaD::AOpyrG* | *ΔgaaD* in N593.20 | This study |
