# Supplementary material for: The pathway intermediate 2‐keto‐3‐deoxy‐L‐galactonate mediates the induction of genes involved in D‐galacturonic acid utilization in Aspergillus niger
Source: FEBS Lett. 2017 May 6;591(10):1408–18. doi: 10.1002/1873-3468.12654 (PMC5488244; doi:10.1002/1873-3468.12654)
Supplement: Supplementary file 3 — Table S2. Primers used in this study. [file FEB2-591-1408-s003.docx]

**Table S2** Primers used in this study.

| **Primer name** | **Sequence (5’ to 3’)** | **Used for** |  |
| --- | --- | --- | --- |
| An02g07710usf | ggggacaactttgtatagaaaagttgTCATGGTTGGAGCGTAGACT | amplification of *gaaA* 5’ flank | ATTB4 site underlined |
| An02g07710usr | ggggactgcttttttgtacaaacttgTGTGATTGCTGTGGTGTAAA | amplification of *gaaA* 5’ flank | ATTB1r site underlined |
| An02g07710dsf | ggggacagctttcttgtacaaagtggATACCTTAGAGAAGCTTGTATG | amplification of *gaaA* 3’ flank | ATTB2r site underlined |
| An02g07710dsr | ggggacaactttgtataataaagttgAGAAGTTCTGTTCAGGCATT | amplification of *gaaA* 3’ flank | ATTB3 site underlined |
| An16g05390usf | ggggacaactttgtatagaaaagttgTCGACGAAGAAGCCGAGGTG | amplification of *gaaB* 5’ flank | ATTB4 site underlined |
| An16g05390usr | ggggactgcttttttgtacaaacttgGGTGTCGGTTGTTTCTGTTCAA | amplification of *gaaB* 5’ flank | ATTB1r site underlined |
| An16g05390dsf | ggggacagctttcttgtacaaagtggGGCGAAGACCATTCTGGAGG | amplification of *gaaB* 3’ flank | ATTB2r site underlined |
| An16g05390dsr | ggggacaactttgtataataaagttgGGAGTAGATGGGCGAGGAATAGGC | amplification of *gaaB* 3’ flank | ATTB3 site underlined |
| An02g07720usf | ggggacaactttgtatagaaaagttgGGGAGCCATTGTGATTGCTG | amplification of *gaaC* 5’ flank | ATTB4 site underlined |
| An02g07720usr | ggggactgcttttttgtacaaacttgGGTTGGAGCGTAGACTCCGG | amplification of *gaaC* 5’ flank | ATTB1r site underlined |
| An02g07720dsf | ggggacagctttcttgtacaaagtggTCCATTGTATCATATAGATTATG | amplification of *gaaC* 3’ flank | ATTB2r site underlined |
| An02g07720dsr | ggggacaactttgtataataaagttgCAGGCTATCGAGTTTATCAC | amplification of *gaaC* 3’ flank | ATTB3 site underlined |
| B1-AoPyrGf | ggggacaagtttgtacaaaaaagcaggctGGATCCTATGGATCTCAGAAC | amplification of *A. oryzae pyrG* gene | ATTB1 site underlined |
| B2-AoPyrGr | ggggaccactttgtacaagaaagctgggtCCGCTGTCGGATCAGGATTA | amplification of *A. oryzae pyrG* gene | ATTB2 site underlined |
| gaaDP1-KpnI | GGGGTACCTCCAAACCGACCGTAAACCA | amplification of *gaaD* 5’ flank | *Kpn*I underlined |
| gaaDP2-XhoI | CCGCTCGAGTGTGTAAGAGGATGTGGTTCAG | amplification of *gaaD* 5’ flank | *Xho*I underlined |
| gaaDP3-HindIII | CCCAAGCTTATCATGGAAATGATCAACGTC | amplification of *gaaD* 3’ flank | *Hind*III underlined |
| gaaDP4-NotI | AAGGAAAAAAGCGGCCGCAACGAGGAGAGGTTGGCGA | amplification of *gaaD* 3’ flank | *Not*I underlined |
| actAP1f | AAGCGTGGTATCCTCACCCT | amplification of actin Northern probe |  |
| actAP2r | TCGTTGCCGATGGTGATGAC | amplification of actin Northern probe |  |
| An14g04280P1f | CGAGCGCATTGGCCGACGCT | amplification of *gatA* Northern probe |  |
| An14g04280P2r | GCATGAGAGTGAAGGCGAAA | amplification of *gatA* Northern probe |  |
| gaaAP1f | TGACGCCCTTCCCGCCGGCTC | amplification of *gaaA* Northern probe |  |
| gaaAP2r | GTTAGAGTGTACACCGGCCC | amplification of *gaaA* Northern probe |  |
| gaaBP1f | CCCGAAGCCATCACCTGGAT | amplification of *gaaB* Northern probe |  |
| gaaBP2r | CCTCCAGAATGGTCTTCGCC | amplification of *gaaB* Northern probe |  |
| gaaCP1f | CCATTCGCAAGCACGCCGTT | amplification of *gaaC* Northern probe |  |
| gaaCP2r | ACGCAACGTATGGGTGGTTT | amplification of *gaaC* Northern probe |  |
| gaaDP1f | CCATGACCCAGCTCCCCAAG | amplification of *gaaD* Northern probe |  |
| gaaDP2r | GCCCAGCTCGGACACCTTGG | amplification of *gaaD* Northern probe |  |
| pgaXP1f | CTGATTACATCCTCTCCGCA | amplification of NRRL3_03144 Northern probe |  |
| pgaXP2r | ATCGGTGTTCTTCGCCTCGT | amplification of NRRL3_03144 Northern probe |  |
| pgxBP1f | TCGCCGTGCTGGACGAACTT | amplification of *pgxB* Northern probe |  |
| pgxBP2r | CGGCGCATCCGTGTTCTGGA | amplification of *pgxB* Northern probe |  |
| abfCP1f | TTGAAGCTCCTTGATCCCAG | amplification of NRRL3_10865 Northern probe |  |
| abfCP2r | ATGGTCCATCCCCGCATATA | amplification of NRRL3_10865 Northern probe |  |
