# Supplementary material for: The pathway intermediate 2‐keto‐3‐deoxy‐L‐galactonate mediates the induction of genes involved in D‐galacturonic acid utilization in Aspergillus niger
Source: FEBS Lett. 2017 May 6;591(10):1408–18. doi: 10.1002/1873-3468.12654 (PMC5488244; doi:10.1002/1873-3468.12654)
Supplement: Supplementary file 4 — Table S3. RNA‐seq analysis of 53 genes of the GaaR‐GaaX panregulon [20] in ∆gaaC and ΔgaaR in GA and in ΔgaaX in d‐fructose. [file FEB2-591-1408-s004.pdf]

Table S3 RNA-seq analysis of 53 genes of the GaaR-GaaX panregulon [20] in *ΔgaaC* and *ΔgaaR* in GA and in *ΔgaaX* in D-fructose. 27 genes belonging to GaaR-GaaX core regulon [20] are written in bold.
Genes with a fold change≥2, and *P* -value≤0.05 or FDR≤0.05 are highlighted.

				This study (FPKM)				Alazi *et al.* , 2016 (FPKM)			Niu *et al.* , 2017 (TPM)			
Gene ID NRRL3	Gene ID CBS513.88	Description[a]	Gene Name	Ref GA[b]	*ΔgaaC* GA	FC *ΔgaaC* GA/Ref GA	*P* -value	*ΔgaaR* GA[b]	FC Ref GA/*ΔgaaR* GA[b]	*P* -value[b]	Ref D-fructose[c]	*ΔgaaX* D-fructose[c]	FC *ΔgaaX* D-fructose/Ref D-fructose[c]	FDR[c]
**NRRL3_00958**	**An14g04280**	**D-galacturonic acid transporter GatA**	***gatA***	888.35	1062.68	1.20	6.95E-02	13.32	66.69	1.54E-03	3.47	524.95	140.38	0.00E+00
**NRRL3_03144**	**An12g07500**	**exo-polygalacturonase**		698.90	3384.63	4.84	1.34E-02	24.27	28.80	1.19E-02	1.36	51.63	32.66	2.60E-272
**NRRL3_05260**	**An02g12450**	**exo-polygalacturonase Pgx28C**	***pgx28C***	99.93	192.85	1.93	9.11E-02	4.10	24.40	6.24E-04	0.95	16.77	15.22	1.83E-144
**NRRL3_05649**	**An02g07720**	**2-keto-3-deoxy-L-galactonate aldolase GaaC**	***gaaC***	5658.32	14.60	0.00	2.88E-04	106.21	53.27	2.98E-04	12.54	2283.77	169.39	0.00E+00
**NRRL3_05650**	**An02g07710**	**D-galacturonic acid reductase GaaA**	***gaaA***	2599.98	6710.72	2.58	1.04E-02	117.53	22.12	1.69E-04	19.92	1515.44	71.84	0.00E+00
**NRRL3_06053**	**An02g02540**	**carbohydrate esterase family 16 protein**		522.81	1301.08	2.49	8.01E-02	22.99	22.75	4.57E-03	2.06	17.62	7.76	5.39E-107
**NRRL3_06890**	**An16g05390**	**L-galactonate dehydratase GaaB**	***gaaB***	11309.00	13990.90	1.24	1.91E-01	344.03	32.87	1.88E-03	47.77	6256.70	121.49	0.00E+00
**NRRL3_08281**	**An03g06740**	**exo-polygalacturonase Pgx28B**	***pgx28B***	200.31	2306.06	11.51	2.82E-02	12.39	16.17	2.62E-02	0.00	2.10	22.95	3.59E-43
**NRRL3_08663**	**An03g01620**	**MFS-type sugar/inositol transporter**		106.09	227.29	2.14	1.71E-01	30.34	3.50	1.25E-01	0.27	5.62	14.28	8.28E-46
**NRRL3_10050**	**An11g01120**	**L-glyceraldehyde reductase GaaD**	***gaaD***	8104.43	7499.78	0.93	5.79E-01	506.79	15.99	7.01E-03	256.41	2732.37	10.16	0.00E+00
**NRRL3_10865**	**An08g01710**	**alpha-N-arabinofuranosidase**		201.62	440.98	2.19	1.92E-01	67.16	3.00	4.04E-02	0.80	11.21	11.92	1.19E-84
**NRRL3_01237**	**An19g00270**	**pectin lyase**		18.95	3.68	0.19	9.55E-03	0.34	55.74	6.03E-04	0.17	1.47	5.25	6.14E-14
**NRRL3_02479**	**An01g10350**	**exo-beta-1,4-galactanase**		137.63	170.01	1.24	5.21E-01	41.24	3.34	1.36E-02	3.48	22.29	6.03	1.37E-179
**NRRL3_05252**	**An02g12505**	**pectin methylesterase**		558.37	3569.08	6.39	2.07E-02	24.68	22.62	4.20E-03	1.06	31.32	25.03	7.10E-189
**NRRL3_07470**	**An04g09690**	**pectin methylesterase**		30.16	12.81	0.42	4.22E-02	4.67	6.46	1.41E-02	0.75	5.72	6.36	3.59E-43
**NRRL3_08325**	**An03g06310**	**pectin methylesterase Pme8A**	***pme8A***	6.54	6.74	1.03	8.79E-01	0.42	15.75	1.18E-02	0.04	0.56	4.10	1.49E-07
**NRRL3_10559**	**An18g04810**	**glycoside hydrolase family 28 protein**		20.00	97.18	4.86	1.19E-02	0.90	22.22	1.26E-02	0.08	3.11	17.32	1.33E-45
NRRL3_00965	An14g04370	pectin lyase Pel1A	*pel1A*	56.54	113.40	2.01	3.58E-01	9.74	5.80	2.12E-04	1.66	3.25	1.84	5.83E-06
NRRL3_04281	An07g00780	MFS-type transporter		90.41	106.00	1.17	5.05E-01	1.86	48.74	7.77E-03	3.11	4.60	#N/A	#N/A
NRRL3_09810	An11g04040	exo-polygalacturonase		10.65	35.99	3.38	7.58E-02	0.34	31.32	9.10E-03	0.01	0.12	#N/A	#N/A
**NRRL3_08194**	**An04g00790**	**Repressor of D-galacturonic acid utilization**	***gaaX***	381.34	529.21	1.39	1.97E-01	20.40	18.70	2.92E-03	15.97	0.00	0.01	2.39E-186
NRRL3_00684	An14g01130	rhamnogalacturonan lyase		5.77	13.23	2.29	2.61E-01	1.03	5.63	9.23E-03	0.02	0.05	#N/A	#N/A
NRRL3_01606	An01g00330	alpha-N-arabinofuranosidase Abf51A	*abf51A*	87.96	111.63	1.27	4.97E-01	59.62	1.48	5.81E-01	1.71	1.94	#N/A	#N/A
NRRL3_02571	An01g11520	endo-polygalacturonase Pga28I	*pga28I*	56.38	59.67	1.06	5.83E-01	6.56	8.59	6.96E-04	0.21	1.24	#N/A	#N/A
NRRL3_02835	An01g14670	endo-polygalacturonase Pga28E	*pga28E*	4.26	13.51	3.17	9.99E-02	2.40	1.78	4.12E-01	0.48	1.15	2.09	1.85E-05
NRRL3_04153	An15g07160	pectin lyase		35.48	19.78	0.56	3.56E-02	37.02	0.96	8.73E-01	18.22	18.64	#N/A	#N/A
NRRL3_04916	An07g08940	carbohydrate esterase family 16 protein		13.41	221.16	16.49	4.37E-02	10.57	1.27	7.42E-01	0.07	0.30	#N/A	#N/A
NRRL3_05859	An02g04900	endo-polygalacturonase Pga28B	*pga28B*	15.10	4.12	0.27	9.36E-02	3.11	4.86	6.74E-02	16.54	18.68	#N/A	#N/A
NRRL3_07094	An16g02730	endo-1,5-alpha-arabinanase		4.57	3.48	0.76	2.43E-01	1.53	2.99	2.66E-02	61.97	61.54	#N/A	#N/A
NRRL3_08805	An05g02440	endo-polygalacturonase Pga28C	*pga28C*	5.26	7.27	1.38	1.85E-01	0.59	8.99	3.65E-02	0.05	0.60	#N/A	#N/A
NRRL3_09811	An11g04030	pectin lyase		0.51	0.11	0.21	6.88E-02	0.00	#DIV/0!	4.77E-03	0.00	0.01	#N/A	#N/A
NRRL3_10643	An18g05940	arabinogalactanase Gan53A	*gan53A*	105.64	67.21	0.64	2.70E-01	29.24	3.61	3.53E-02	1.74	3.85	2.06	1.07E-07
NRRL3_11738	An06g00290	beta-galactosidase		28.91	319.96	11.07	4.60E-02	9.08	3.19	3.55E-02	0.35	0.93	2.34	4.28E-07
NRRL3_00502	An09g06200	hypothetical protein		14.07	41.41	2.94	1.16E-01	39.06	0.36	6.88E-02	0.93	12.38	9.36	1.08E-32
**NRRL3_00660**	**An14g00860**	**carboxylesterase**		74.22	825.36	11.12	4.58E-02	16.04	4.63	5.24E-02	0.21	1.87	6.98	1.47E-31
NRRL3_00957	An14g04260	B3/B4 domain-containing protein		7.87	13.03	1.66	2.87E-01	0.20	39.33	1.62E-01	1.53	18.56	10.05	2.46E-64
NRRL3_01073	An14g05840	O-methyltransferase, COMT-type		3.22	11.45	3.55	1.39E-02	6.54	0.49	3.99E-01	0.54	5.57	7.16	1.06E-21
**NRRL3_01127**	**An14g06500**	**dihydroxyacetone kinase**		584.25	203.94	0.35	1.55E-02	270.64	2.16	1.70E-02	18.90	100.64	4.98	1.61E-102
**NRRL3_01398**	**An13g02090**	**MFS-type transporter**		26.10	96.31	3.69	1.69E-02	3.01	8.69	3.81E-02	2.81	13.10	4.16	5.62E-30
NRRL3_02770	An01g13880	MFS-type transporter		3.71	6.43	1.73	9.57E-02	6.87	0.54	2.43E-01	0.87	4.07	4.06	1.57E-20
NRRL3_03291	An12g05600	heterokaryon incompatibility protein		0.80	6.04	7.60	6.39E-02	0.00	#DIV/0!	3.25E-01	0.00	6.78	82.98	6.48E-78
NRRL3_03292	An12g05590	carboxylesterase		0.25	1.72	6.88	3.30E-01	1.53	0.16	2.64E-01	0.18	1.86	5.26	1.75E-09
**NRRL3_03342**	**An12g04990**	**short-chain dehydrogenase/reductase**		151.58	706.28	4.66	1.05E-02	22.32	6.79	7.20E-03	0.81	14.46	13.51	2.10E-62
NRRL3_03467	An12g03550	MFS-type transporter		4.91	92.55	18.85	2.61E-02	302.24	0.02	3.45E-03	1.13	6.73	5.05	4.03E-28
**NRRL3_06244**	**An02g00140**	**glycoside hydrolase family 43 protein**		80.90	137.44	1.70	1.81E-01	5.13	15.77	3.00E-03	0.81	22.19	23.46	8.13E-193
NRRL3_07382	An16g00540	alpha-L-fucosidase		2.29	8.06	3.53	4.41E-02	1.47	1.56	6.90E-01	0.04	0.67	7.07	5.92E-14
NRRL3_08499	An03g03960	uncharacterized protein		13.64	45.86	3.36	6.05E-03	37.95	0.36	2.56E-01	0.48	4.58	6.71	3.84E-21
**NRRL3_08833**	**n.a.**	**hypothetical protein**		4.29	1.87	0.44	2.27E-02	0.42	10.33	1.16E-02	0.31	2.02	4.52	1.58E-12
NRRL3_09862	An11g03510	hypothetical protein		0.43	0.20	0.45	5.62E-01	0.00	#DIV/0!	2.64E-01	0.00	0.93	11.05	4.32E-18
**NRRL3_09863**	**An11g03500**	**alpha-hydroxy acid dehydrogenase, FMN-dependent**		59.53	64.98	1.09	2.85E-01	0.62	96.80	4.13E-03	0.82	165.84	160.07	0.00E+00
**NRRL3_10558**	**An18g04800**	**alpha-L-rhamnosidase**		17.04	109.06	6.40	3.54E-02	1.80	9.46	2.67E-02	0.35	3.85	9.09	2.19E-61
**NRRL3_11054**	**An08g04040**	**MFS-type sugar/inositol transporter**		693.37	4713.62	6.80	8.89E-03	285.86	2.43	5.55E-03	6.62	31.89	4.57	2.57E-126
NRRL3_11710	An06g00620	MFS-type sugar/inositol transporter		341.35	1977.10	5.79	2.76E-02	680.84	0.50	4.40E-03	2.86	25.29	8.05	1.39E-118

[a] Descriptions were obtained from manual annotation (manuscript in preparation).
[b] Data published by Alazi *et al.* [19]
[c] Data published by Niu *et al.* [20]
