# Supplementary material for: The pathway intermediate 2‐keto‐3‐deoxy‐L‐galactonate mediates the induction of genes involved in D‐galacturonic acid utilization in Aspergillus niger
Source: FEBS Lett. 2017 May 6;591(10):1408–18. doi: 10.1002/1873-3468.12654 (PMC5488244; doi:10.1002/1873-3468.12654)
Supplement: Supplementary file 5 — Table S4. RNA‐seq analysis of pectinases in ∆gaaC and ∆gaaR in GA and in ∆gaaX in d‐fructose. [file FEB2-591-1408-s005.pdf]

Table S4 RNA-seq analysis of pectinases in *ΔgaaC* and *ΔgaaR* in GA and in *ΔgaaX* in D-fructose.
Genes with a fold change≥2, and *P*-value≤0.05 or FDR≤0.05 are highlighted.

				This study (FPKM)				Alazi *et al.*, 2016 (FPKM)			Niu *et al.*, 2017 (TPM)			
Gene ID NRRL3	Gene ID CBS513.88	Description[a]	Gene Name	Ref GA[b]	*ΔgaaC* GA	FC *ΔgaaC* GA/Ref GA	*P*-value	*ΔgaaR* GA[b]	FC Ref GA/*ΔgaaR* GA[b]	*P*-value[b]	Ref D-fructose[c]	*ΔgaaX* D-fructose[c]	FC *ΔgaaX* D-fructose/Ref D-fructose[c]	FDR[c]
NRRL3_03144	An12g07500	exo-polygalacturonase		698.90	3384.63	4.84	1.34E-02	24.27	28.80	1.19E-02	1.36	51.63	32.66	2.60E-272
NRRL3_09810	An11g04040	exo-polygalacturonase		10.65	35.99	3.38	7.58E-02	0.34	31.32	9.10E-03	0.01	0.12	#N/A	#N/A
NRRL3_08281	An03g06740	exo-polygalacturonase Pgx28B	*pgx28B*	200.31	2306.06	11.51	2.82E-02	12.39	16.17	2.62E-02	0.00	2.10	22.95	3.59E-43
NRRL3_05260	An02g12450	exo-polygalacturonase Pgx28C	*pgx28C*	99.93	192.85	1.93	9.11E-02	4.10	24.40	6.24E-04	0.95	16.77	15.22	1.83E-144
NRRL3_06053	An02g02540	carbohydrate esterase family 16 protein		522.81	1301.08	2.49	8.01E-02	22.99	22.75	4.57E-03	2.06	17.62	7.76	5.39E-107
NRRL3_04916	An07g08940	carbohydrate esterase family 16 protein		13.41	221.16	16.49	4.37E-02	10.57	1.27	7.42E-01	0.07	0.30	#N/A	#N/A
NRRL3_08325	An03g06310	pectin methylesterase Pme8A	*pme8A*	6.54	6.74	1.03	8.79E-01	0.42	15.75	1.18E-02	0.04	0.56	4.10	1.49E-07
NRRL3_07470	An04g09690	pectin methylesterase		30.16	12.81	0.42	4.22E-02	4.67	6.46	1.41E-02	0.75	5.72	6.36	3.59E-43
NRRL3_05252	An02g12505	pectin methylesterase		558.37	3569.08	6.39	2.07E-02	24.68	22.62	4.20E-03	1.06	31.32	25.03	7.10E-189
NRRL3_02571	An01g11520	endo-polygalacturonase Pga28I	*pga28I*	56.38	59.67	1.06	5.83E-01	6.56	8.59	6.96E-04	0.21	1.24	#N/A	#N/A
NRRL3_04000	An15g05370	endo-polygalacturonase Pga28II	*pga28II*	1.67	0.11	0.06	1.01E-01	0.68	2.46	3.70E-01	0.29	1.06	#N/A	#N/A
NRRL3_06782	An16g06990	endo-polygalacturonase Pga28A	*pga28A*	0.70	1.66	2.38	5.66E-01	1.24	0.56	5.76E-01	5.97	6.72	#N/A	#N/A
NRRL3_05859	An02g04900	endo-polygalacturonase Pga28B	*pga28B*	15.10	4.12	0.27	9.36E-02	3.11	4.86	6.74E-02	16.54	18.68	#N/A	#N/A
NRRL3_08805	An05g02440	endo-polygalacturonase Pga28C	*pga28C*	5.26	7.27	1.38	1.85E-01	0.59	8.99	3.65E-02	0.05	0.60	#N/A	#N/A
NRRL3_00263	An09g03260	endo-polygalacturonase Pga28D	*pga28D*	2.55	4.44	1.74	2.14E-02	2.36	1.08	7.98E-01	0.93	1.07	#N/A	#N/A
NRRL3_02835	An01g14670	endo-polygalacturonase Pga28E	*pga28E*	4.26	13.51	3.17	9.99E-02	2.40	1.78	4.12E-01	0.48	1.15	2.09	1.85E-05
NRRL3_00965	An14g04370	pectin lyase Pel1A	*pel1A*	56.54	113.40	2.01	3.58E-01	9.74	5.80	2.12E-04	1.66	3.25	1.84	5.83E-06
NRRL3_08767	An03g00190	pectin lyase Pel1B	*pel1B*	7.86	4.42	0.56	1.29E-01	15.52	0.51	3.67E-02	5.07	5.03	#N/A	#N/A
NRRL3_09811	An11g04030	pectin lyase		0.51	0.11	0.21	6.88E-02	0.00	#DIV/0!	4.77E-03	0.00	0.01	#N/A	#N/A
NRRL3_01237	An19g00270	pectin lyase		18.95	3.68	0.19	9.55E-03	0.34	55.74	6.03E-04	0.17	1.47	5.25	6.14E-14
NRRL3_04153	An15g07160	pectin lyase Pel1C	*pel1C*	35.48	19.78	0.56	3.56E-02	37.02	0.96	8.73E-01	18.22	18.64	#N/A	#N/A
NRRL3_06359	An10g00870	pectate lyase Ply1A	*ply1A*	2.27	3.65	1.61	3.73E-01	4.63	0.49	3.54E-01	0.15	0.13	#N/A	#N/A
NRRL3_07469	An04g09700	xylogalacturonase		2.82	2.20	0.78	2.58E-01	1.91	1.48	6.50E-01	0.17	0.34	#N/A	#N/A
NRRL3_02832	An01g14650	glycoside hydrolase family 28 protein		1.49	12.95	8.69	1.21E-02	3.46	0.43	2.83E-02	0.18	0.25	#N/A	#N/A
NRRL3_08631	An03g02080	alpha-L-rhamnosidase Rha28B	*rgx28B*	0.13	2.24	17.19	8.32E-02	0.34	0.38	4.01E-01	0.00	0.02	#N/A	#N/A
NRRL3_10559	An18g04810	glycoside hydrolase family 28 protein		20.00	97.18	4.86	1.19E-02	0.90	22.22	1.26E-02	0.08	3.11	17.32	1.33E-45
NRRL3_09126	An12g00950	endo-rhamnogalacturonase Rhg28A	*rhg28A*	2.25	4.09	1.82	8.87E-02	2.57	0.88	6.35E-01	1.38	1.58	#N/A	#N/A
NRRL3_00953	An14g04200	endo-rhamnogalacturonase Rhg28B	*rhg28B*	5.24	5.99	1.14	7.67E-01	6.89	0.76	5.26E-01	3.47	5.05	#N/A	#N/A
NRRL3_11790	An06g02070	endo-rhamnogalacturonase		0.00	0.43	NA	NA	0.53	0.00	NA	0.22	0.06	#N/A	#N/A
NRRL3_09623	An11g06320	endo-rhamnogalacturonase		0.00	0.00	NA	NA	0.00	#DIV/0!	NA	0.00	0.01	#N/A	#N/A
NRRL3_09450	An11g08700	endo-rhamnogalacturonase		1.75	4.34	2.48	3.39E-02	9.38	0.19	1.10E-03	0.17	0.30	#N/A	#N/A
NRRL3_04303	An07g01000	rhamnogalacturonan hydrolase		0.00	0.00	NA	NA	0.00	#DIV/0!	NA	0.00	0.00	#N/A	#N/A
NRRL3_00684	An14g01130	rhamnogalacturonan lyase		5.77	13.23	2.29	2.61E-01	1.03	5.63	9.23E-03	0.02	0.05	#N/A	#N/A
NRRL3_10115	An11g00390	rhamnogalacturonan lyase		5.91	15.95	2.70	1.75E-01	3.66	1.61	6.02E-02	0.65	0.81	#N/A	#N/A
NRRL3_00169	An09g02160	rhamnogalacturonan acetylesterase		8.03	73.25	9.12	1.66E-01	16.53	0.49	2.55E-02	0.45	0.68	#N/A	#N/A
NRRL3_07501	An04g09360	carbohydrate esterase family 12 protein		17.42	87.29	5.01	4.60E-02	28.91	0.60	9.59E-02	1.31	1.13	#N/A	#N/A
NRRL3_00839	An14g02920	glycoside hydrolase family 105 protein		3.61	22.81	6.32	5.98E-03	27.70	0.13	6.70E-02	5.00	5.09	#N/A	#N/A
NRRL3_01038	An14g05340	glycoside hydrolase family 105 protein		0.12	0.00	0.00	4.23E-01	0.30	0.40	3.24E-01	0.33	0.37	#N/A	#N/A
NRRL3_01606	An01g00330	alpha-N-arabinofuranosidase Abf51A	*abf51A*	87.96	111.63	1.27	4.97E-01	59.62	1.48	5.81E-01	1.71	1.94	#N/A	#N/A
NRRL3_03768	An15g02300	alpha-N-arabinofuranosidase Abf54B	*abf54B*	240.98	1543.35	6.40	1.14E-01	445.99	0.54	1.94E-02	0.81	3.67	3.92	5.05E-19
NRRL3_10865	An08g01710	alpha-N-arabinofuranosidase		201.62	440.98	2.19	1.92E-01	67.16	3.00	4.04E-02	0.80	11.21	11.92	1.19E-84
NRRL3_00064	An09g00880	alpha-N-arabinofuranosidase		0.00	0.00	NA	NA	0.00	#DIV/0!	NA	0.00	0.00	#N/A	#N/A
NRRL3_00092	An09g01190	endo-1,5-alpha-arabinanase Abn43A	*abn43A*	26.33	235.56	8.95	3.25E-01	287.90	0.09	4.59E-04	0.02	0.01	#N/A	#N/A
NRRL3_06141	An02g01400	endo-1,5-alpha-arabinanase		3.88	7.59	1.95	1.13E-02	7.47	0.52	4.52E-02	6.70	6.61	#N/A	#N/A
NRRL3_05407	An02g10550	endo-1,5-alpha-arabinanase		103.20	702.79	6.81	1.45E-02	1986.46	0.05	1.14E-01	9.99	15.44	#N/A	#N/A
NRRL3_07094	An16g02730	endo-1,5-alpha-arabinanase		4.57	3.48	0.76	2.43E-01	1.53	2.99	2.66E-02	61.97	61.54	#N/A	#N/A
NRRL3_00007	An09g00120	feruloyl esterase FaeA	*faeA*	7.83	12.62	1.61	1.99E-01	7.41	1.06	9.60E-01	0.06	0.00	#N/A	#N/A
NRRL3_02931	An12g10390	feruloyl esterase FaeB	*faeB*	4.17	16.38	3.93	3.08E-02	4.34	0.96	7.78E-01	8.98	9.92	#N/A	#N/A
NRRL3_06870	An16g05690	lipase, class 3		0.00	0.00	NA	NA	0.00	#DIV/0!	NA	0.02	0.03	#N/A	#N/A
NRRL3_08993	An12g02550	feruloyl esterase		0.58	0.00	0.00	4.23E-01	0.15	4.00	5.43E-01	0.00	0.02	#N/A	#N/A
NRRL3_02630	An01g12150	beta-galactosidase Lac35B	*lac35B*	172.89	1259.38	7.28	3.28E-02	228.36	0.76	3.16E-01	1.59	2.20	#N/A	#N/A
NRRL3_02479	An01g10350	exo-beta-1,4-galactanase		137.63	170.01	1.24	5.21E-01	41.24	3.34	1.36E-02	3.48	22.29	6.03	1.37E-179
NRRL3_11738	An06g00290	exo-beta-1,4-galactanase		28.91	319.96	11.07	4.60E-02	9.08	3.19	3.55E-02	0.35	0.93	2.34	4.28E-07
NRRL3_04568	An07g04420	exo-beta-1,4-galactanase		0.23	9.58	41.63	7.17E-03	0.84	0.27	1.19E-01	0.03	0.04	#N/A	#N/A
NRRL3_01071	An14g05820	beta-galactosidase		0.75	8.06	10.74	2.90E-02	3.60	0.21	5.01E-02	0.02	0.04	#N/A	#N/A
NRRL3_11773	An08g10780	exo-beta-1,4-galactanase	*gbgA*	115.29	125.86	1.09	7.82E-01	52.13	2.21	6.92E-02	3.35	3.15	#N/A	#N/A
NRRL3_10643	An18g05940	arabinogalactanase Gan53A	*gan53A*	105.64	67.21	0.64	2.70E-01	29.24	3.61	3.53E-02	1.74	3.85	2.06	1.07E-07
#N/A	An16g06590	#N/A		#N/A	#N/A	#N/A	#N/A	#N/A	#N/A	#N/A	#N/A	#N/A	#N/A	#N/A

[a] Descriptions were obtained from manual annotation (manuscript in preparation).
[b] Data published by Alazi *et al.* [19]
[c] Data published by Niu *et al.* [20]
